# Percutaneous kyphoplasty with or without temporary unipedicle screw reduction: A retrospective comparative study of osteoporotic vertebral fractures

**DOI:** 10.1007/s00132-016-3235-z

**Published:** 2016-03-03

**Authors:** T. Zhu, Y. Tian, F. Zhou, L. Shang, Y. Guo, Y. Lv

**Affiliations:** Orthopedic Trauma, Peking University Third Hospital, No. 49 North Garden Road, HaiDian District, 100191 Beijing, China

**Keywords:** Osteoporotic vertebral compression fractures, Reduction, Fractures, bone, Pedicle screws, Spine, Osteoporotische Wirbelkörperkompressionsfrakturen, Reposition, Knochenfrakturen, Pedikelschrauben, Wirbelsäule

## Abstract

**Background:**

Temporary unipedicle screw reduction with percutaneous kyphoplasty (TUSR-PKP) is a relatively new method for managing osteoporotic vertebral compression fractures (OVCFs). A clinical retrospective comparative study was conducted to verify whether TUSR-PKP was noninferior to simple PKP regarding the management of OVCFs.

**Methods:**

A total of 38 consecutive patients who sustained OVCFs without neurological deficits and had undergone surgeries in our hospital from June 2012 to January 2014 were included in the study: 24 patients underwent simple PKP (control group) and the other 14 patients underwent TUSR-PKP (treatment group). All 38 patients were asked to participate in a long-term (>1 year) follow-up. Visual analog scale (VAS) pain scores and Oswestry Disability Index (ODI) were recorded, and the Cobb angles and the vertebral body heights were measured on the lateral radiographs before surgery and on day 1, as well as 1, 3, 6, and 12 months after surgery.

**Results:**

The patients in the treatment group had better vertebral height gain and greater improvement on ODI compared with the control group (p < 0.05). The VAS scores of the two groups were similar at all points until the end of the 1‑year follow-up period. Two patients from the treatment group and 5 patients from the control group had cement leakage. In the control group, 3 patients suffered adjacent or nonadjacent vertebra fractures.

**Conclusion:**

TUSR-PKP is a safe and effective surgical option for OVCFs. Compared with simple PKP, TUSR-PKP provided at least equal results for OVCFs. Moreover, during the postsurgery observations, TUSR-PKP showed potential advantages including vertebral height gain, ODI improvement, and fewer subsequent refractures.

## Zusammenfassung

**Hintergrund:**

Die temporäre Reposition mithilfe unilateraler Pedikelschrauben in Kombination mit der perkutanen Kyphoplastie (TUSR-PKP) ist ein relativ neues Verfahren zur Behandlung osteoporotischer Wirbelkörperkompressionsfrakturen (OVCF). In einer retrospektiven klinischen Vergleichsstudie wurde untersucht, ob die TUSR-PKP der alleinigen PKP in der Behandlung von OVCF nicht unterlegen ist.

**Methoden:**

In die Studie wurden insgesamt 38 konsekutive Patienten mit OVCF eingeschlossen, die keine neurologischen Einschränkungen aufwiesen und in unserer Klinik zwischen Juni 2012 und Januar 2014 einem chirurgischen Eingriff unterzogen wurden. Bei 24 Patienten wurde lediglich eine PKP durchgeführt (Kontrollgruppe), 14 Patienten erhielten eine TUSR-PKP (Behandlungsgruppe). Alle 38 Patienten wurden gebeten, an einer Langzeitnachverfolgung (> 1 Jahr) teilzunehmen. Schmerzintensitäten auf einer visuellen Analogskala (VAS) und der Oswestry Disability Index (ODI) wurden dokumentiert. Cobb-Winkel und die Wirbelkörperhöhe wurden auf lateralen Röntgenaufnahmen vor dem Eingriff sowie 1 Tag, 1, 3, 6 und 12 Monate danach bestimmt.

**Ergebnisse:**

Bei den Patienten der Behandlungsgruppe war im Vergleich zur Kontrollgruppe eine größere Zunahme der Wirbelkörperhöhe und eine stärkere Verbesserung des ODI zu verzeichnen (p < 0,05). Die VAS-Werte waren in den beiden Gruppen zu allen Zeitpunkten bis zum Ende der 1‑jährigen Nachverfolgungsphase vergleichbar. Bei 2 Patienten der Behandlungs- und bei 5 Patienten der Kontrollgruppe kam es zu einem Zementaustritt. In der Kontrollgruppe ereigneten sich bei 3 Patienten Frakturen an angrenzenden oder nichtangrenzenden Wirbelkörpern.

**Schlussfolgerung:**

Die TUSR-PKP ist eine sichere und wirksame chirurgische Option in der Behandlung von OVCF. Im Vergleich zur alleinigen PKP werden mit der TUSR-PKP bei OVCF mindestens gleichwertige Ergebnisse erzielt. Darüber hinaus zeigten sich in der postoperativen Nachverfolgung potenzielle Vorteile des neuen Verfahrens, zu denen die Zunahme der Wirbelkörperhöhe, die ODI-Verbesserung und eine geringere Zahl späterer Refrakturen zählen.

## Introduction

Percutaneous kyphoplasty (PKP) is a minimally invasive surgical treatment and has become increasingly popular for the treatment of painful osteoporotic vertebral compression fractures (OVCFs) refractory to conservative therapy. Despite excellent clinical results on pain relief and function improvement after PKP, there are still some disadvantages such as unsatisfactory reduction of fracture, bone cement leakage, and postoperative complications including the adjacent and nonadjacent vertebra fractures or the injured vertebra refractures, which always lead to severe pain and dysfunction [1–3].

To achieve better reduction and to minimize complications, we invented a new minimally invasive method—temporary unilateral pedicle screw reduction with percutaneous kyphoplasty (TUSR-PKP)—where the following were performed prior to traditional PKP: (1) implantation of two Schanz pedicle screws unilaterally in the pedicle of adjacent upper/lower segments percutaneously, (2) connection of the Schanz screws to a longitudinal rod, and (3) use of a distraction tool to achieve satisfactory vertebra reduction. It is speculated that temporary percutaneous pedicle screw reduction with PKP (TUSR-PKP) could be a good choice for the treatment of OVCFs. Hence, a retrospective study comparing the simple PKP (the control group) with TUSR-PKP (the treatment group) was conducted. Our hypothesis was that the TUSR-PKP is noninferior to simple PKP in managing OVCFs.

## Patients and methods

### Selection of patients

In this retrospective study, consecutive patients (*n* = 140) who received treatment for OVCFs in our hospital between January 2012 and January 2014 were initially screened. The study inclusion criteria were as follows: (1) patients older than 50 years; (2) clear trauma history within a week (e. g., fall, road traffic accident); (3) no symptoms or physical findings of nerve damage; (4) a single-level vertebral compression fracture with obvious anterior height loss; (5) severe back pain with preoperative visual analog scale (VAS) score ≥ 7; and (6) patients who had been followed up for at least 1 year postsurgery. The study exclusion criteria were as follows: (1) patients who could not tolerate surgery; (2) pathologic fracture; (3) multiple-level fractures; (4) patients who failed to follow-up for at least 1 year; and (5) no willingness to participate in this study. As a result, 38 patients were enrolled in the study. The causes of the injury were falls to the ground (*n* = 33) and traffic accidents (*n* = 5). Prior to surgery, detailed information about both TUSR-PKP and simple PKP approaches, as well as the additional cost and potential benefits associated with TUSR-PKP were provided to the selected 38 patients and their families; afterwards, they were asked to make an informed decision. Thus, based on the surgical procedures they preferred, the participants were divided into two groups: the control group (simple PKP, *n* = 24) and the treatment group (TUSR-PKP, *n* = 14). The demographic characteristics of the patients are presented in Tab. 1. There was no significant difference between the two groups with respect to most demographic parameters; hence, the study protocol was approved by the Ethics Committees of our hospital, and then we requested written informed consent from all participants.

**Tab. 1:** Demographic characteristics of the two groups

	Treatment group	Control group	P value
Patients (n)	14	24	
Age (years)^a^	63.93 ± 6.94	67.63 ± 9.54	> 0.05
Male:female ratio	3:11	5:19	> 0.05
*Level of fracture*			
T5		1	
T7		1	
T8		1	
T11	1	1	
T12	4	4	
L1	7	7	
L2	0	4	
L3	1	3	
L4	1	2	
Duration of follow-up (months)^a^	13.64 ± 1.28	14.54 ± 2.43	> 0.05

^a^Values represented as mean ± standard deviation

### Surgical procedures

**Fig. 1:**
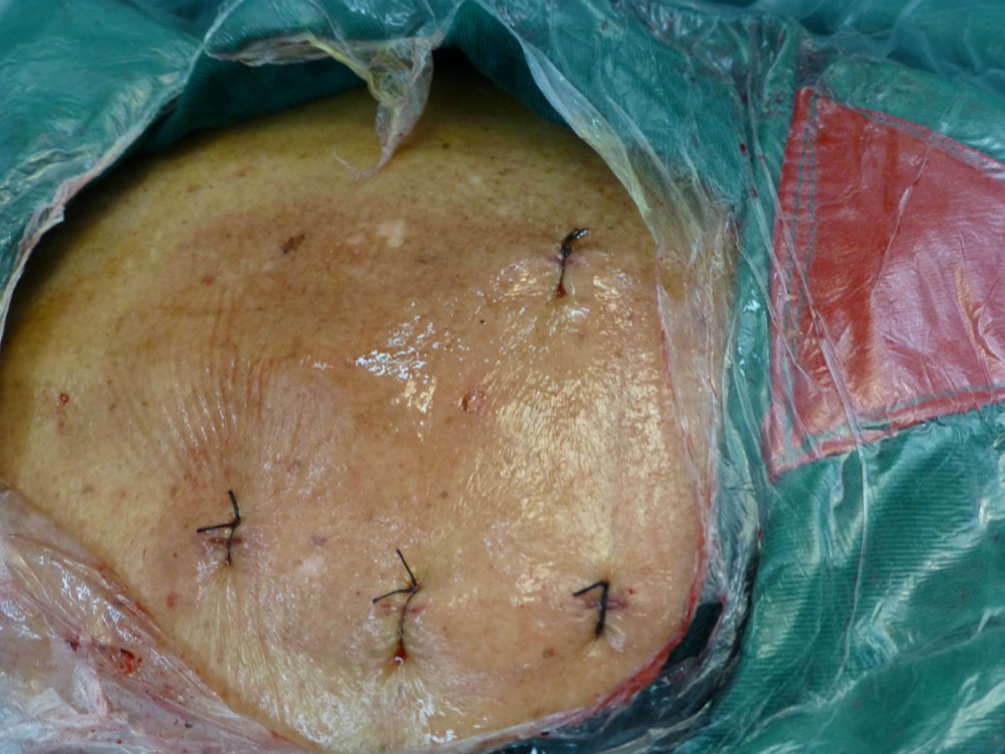
Skin incision design for percutaneous pedicle screw implantation and percutaneous kyphoplasty

**Fig. 2:**
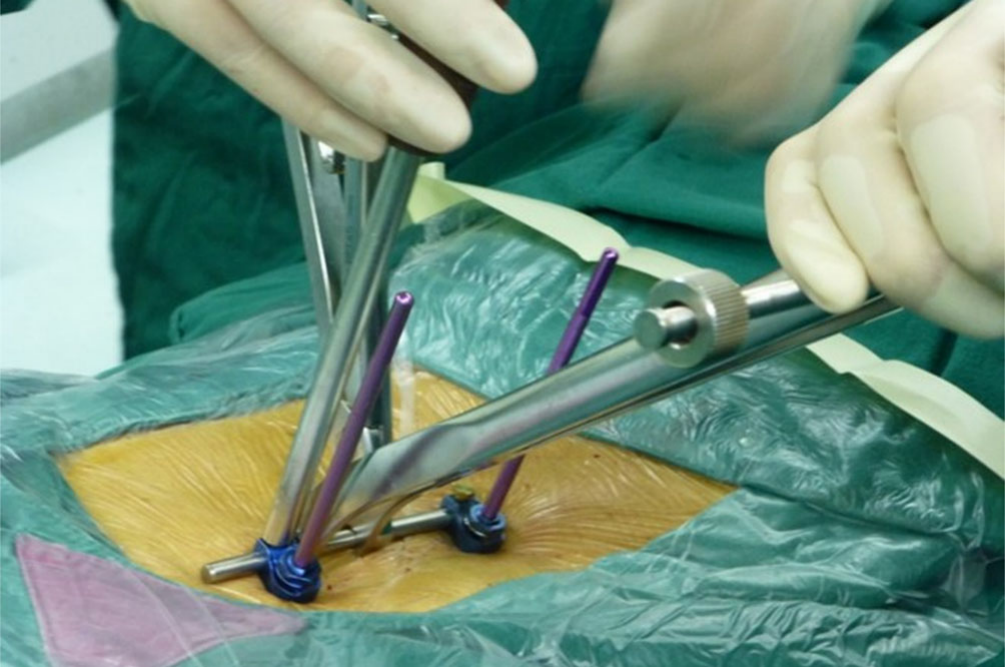
Temporary unilateral pedicle screw reduction with connecting rod

**Fig. 3:**
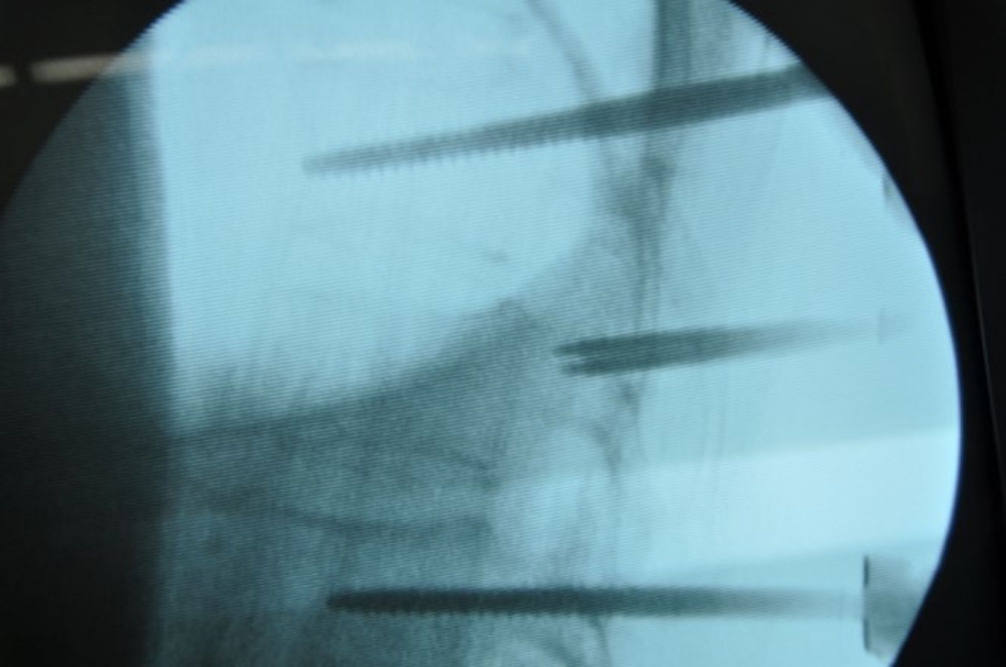
Intraoperative X‑ray showed adequate reduction through Schanz screws

- Before surgery, patients in the treatment group were placed in the prone position under general anesthesia with endotracheal intubation and the position of the pedicle of the injured vertebra and the unilateral adjacent upper/lower segments were marked on the skin using G‑arm fluoroscopy.
- After disinfection and placement of sterile drapes, a skin incision of about 3 mm was made unilaterally (right or left, depending on which side was seriously injured) in the pedicle of adjacent upper/lower segments. Two Schanz pedicle screws (Synthes, Switzerland) were then implanted percutaneously under G‑arm fluoroscopy (Fig. 1) and a longitudinal rod was then connected with the Schanz screws outside of the skin.

- Next, the distraction reposition of the fractured vertebra was performed along the longitudinal direction of the connecting rod using a distraction tool (Fig. 2). Then the Schanz screws were rotated to restore the anterior collapsed wall. Finally, the screws were tightened after satisfactory vertebra reduction (Fig. 3).

- Subsequently, PKP was performed. A trocar of 5 mm diameter was inserted into the injured vertebral body in the bilateral side of the pedicle. Bone cement (PMMA, Italy; GeneX, England) was injected into the fractured vertebra after balloon (KYPHON®) expansion. Ten minutes after the hardening of cement, the Schanz pedicle screws were removed and the surgical wound was washed and sealed.

However, the patients in the control group only underwent traditional PKP. In this study, all surgical procedures were performed by the same team of experienced surgeons and the postoperative care was also managed in the same manner for both groups.

### Clinical data

**Fig. 4:**
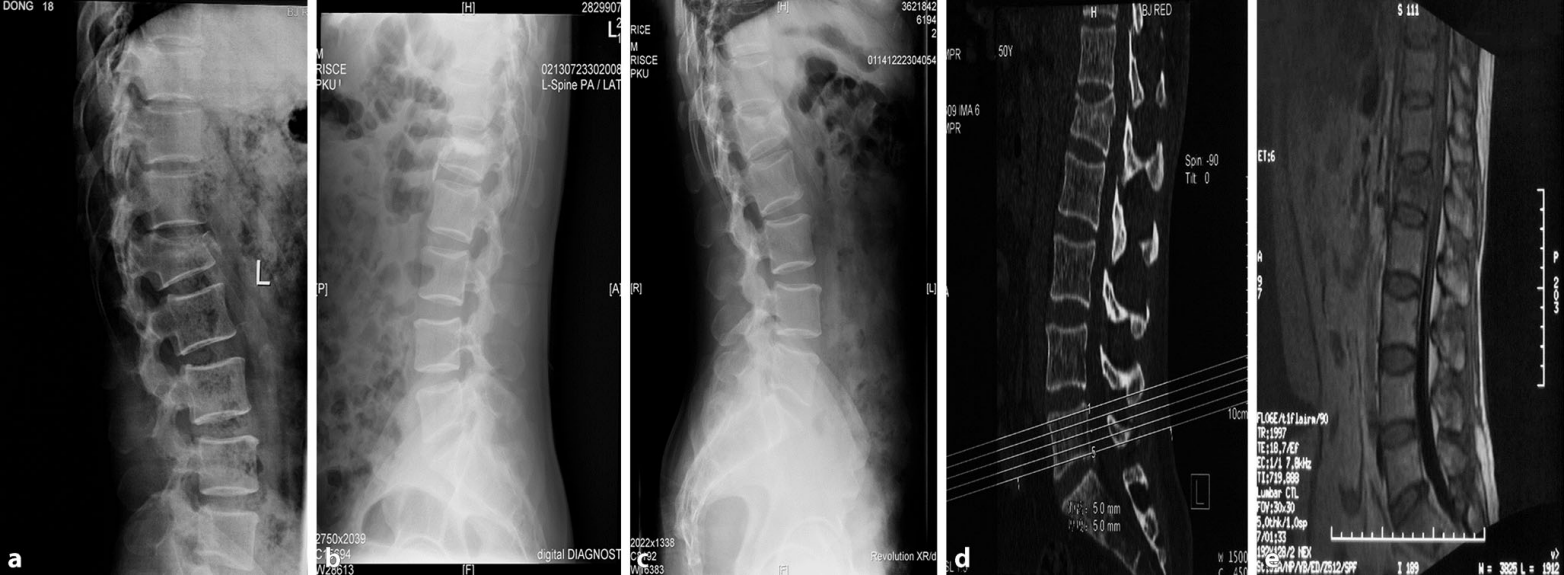
Radiograph (magnetic resonance imaging [*MRI*], computed tomography [*CT*], and X‑ray) images of typical case treated with simple percutaneous kyphoplasty: **a** perioperative X‑ray, **b** postoperative X‑ray, **c** postoperative 17-month follow-up X‑ray, **d** perioperative CT, **e** perioperative MRI

**Fig. 5:**
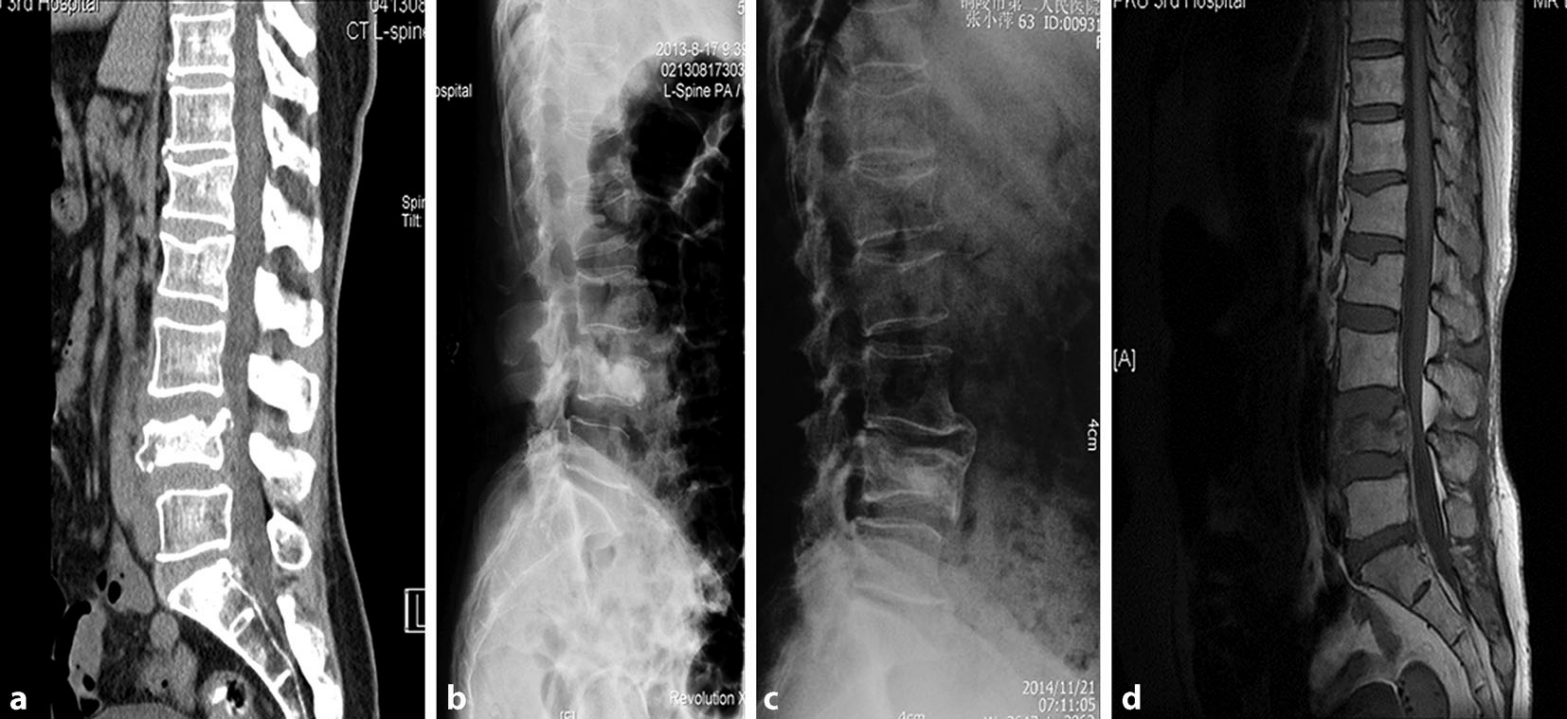
Radiograph (magnetic resonance imaging [*MRI*], computed tomography [*CT*], and X‑ray) images of typical case treated with temporary unilateral pedicle screw reduction with percutaneous kyphoplasty: **a** perioperative CT, **b** postoperative X‑ray, **c** postoperative 15-month follow-up X‑ray, **d** perioperative MRI

Clinical data consisting of surgery duration, amount of blood loss, and volume of bone cement injected for both groups were recorded. All perioperative medical or surgical complications were reported and all patients were evaluated on day 1 as well as 1, 3, 6, and 12 months after surgery. The VAS score for pain and the Oswestry Disability Index (ODI), a questionnaire on quantify functional disability related to back pain, were assessed. All imaging data (radiography, computed tomography [CT], and/or magnetic resonance image [MRI]) were evaluated. The local kyphosis angle (Cobb angle, formed by the inferior end plate of the intact vertebra cephalad to the fracture and the superior end plate of the intact vertebra caudad to the fracture) and the central and anterior vertebral body heights (the measured central and anterior heights divided by that of the intact posterior wall) were measured on lateral plain radiographs of the standing position by two physicians who were not involved in patient care after surgery. A CT scan was used to evaluate bone cement leakage, vertebral refracture, and adjacent and nonadjacent vertebral fractures at least once during the follow-up. All the procedural complications were recorded and all the data were recorded by other physicians who were not involved during surgery to avoid the potential biases (Figs. 4 and 5).

### Statistical analysis

All the data are recorded as mean ± standard deviation. Data analysis was performed using SPSS 22.0;  P < 0.05 was considered statistically significant.

## Results

The results showed that the difference was insignificant between the controlled and treatment groups on VAS, ODI (*p* > 0.05) before surgery, but the values of anterior vertebral body height, central vertebral body height, and local Cobb angle differed significantly. It was assumed that the condition of the back pain and physical function were similar between the two groups but vertebral compression was worse in the treatment group.

### VAS, ODI scores, and vertebral body height

In the treatment group, the VAS score was lower immediately after surgery (2.00 ± 1.58; *p* < 0.005) and at the end of the follow-up period (0.64 ± 0.74; *p* < 0.005) compared to before surgery (7.89 ± 1.57). The ODI score also was lower immediately after surgery (0.34 ± 0.16; *p* < 0.005) and at the end of the follow-up period (0.10 ± 0.04; *p* < 0.005) compared to before surgery (0.73 ± 0.18). On the other hand, the anterior vertebral body height increased from 0.56 ± 0.10 before surgery to 0.73 ± 0.11 (*p* < 0.005) immediately after surgery and to 0.68 ± 0.11 (*p* < 0.005) at the end of the follow-up period. The central vertebral body height also increased from 0.60 ± 0.07 before surgery to 0.78 ± 0.11 (*p* < 0.005) immediately after surgery and to 0.67 ± 0.13 (*p* < 0.005) at the end of the follow-up period. The local Cobb angle decreased from 17.97 ± 4.38° before surgery to 10.25 ± 4.51° (*p* < 0.005) immediately after surgery and was 13.72 ± 4.42° (*p* < 0.005) at the end of the follow-up period. By adopting the method of the Bonferroni adjustment, *p* < 0.01 is considered statistically significant. Therefore, we observed significant differences (*p* < 0.005) between both pre- and postoperative data and between preoperative and at the 1‑year follow-up values of all parameters. All results for the treatment group are presented in Tab. 2.

**Tab. 2:** Clinical parameters from initial evaluation to last follow-up in the treatment group

Parameters	Preop	Postop	1 Month	3 Months	6 Months	1 Year
VAS^a^	7.89 ± 1.57	2.00 ± 1.58	1.10 ± 0.57	0.60 ± 0.70	0.60 ± 0.89	0.64 ± 0.74
ODI	0.73 ± 0.18	0.34 ± 0.16	0.22 ± 0.14	0.17 ± 0.07	0.16 ± 0.15	0.10 ± 0.04
Anterior VB height	0.56 ± 0.10	0.73 ± 0.11	0.66 ± 0.06	0.65 ± 0.11	0.60 ± 0.06	0.68 ± 0.11
Central VB height	0.60 ± 0.07	0.78 ± 0.11	0.73 ± 0.11	0.72 ± 0.07	0.68 ± 0.03	0.67 ± 0.13
Local Cobb angle (°)	17.97 ± 4.38	10.25 ± 4.51	13.23 ± 3.59	14.46 ± 5.90	15.46 ± 2.88	13.72 ± 4.42

*VAS* visual analog scale, *ODI* Oswestry Disability Index, *Preop* preoperative, *Postop* postoperative, *VB* vertebral body

^a^Values represented as mean ± standard deviation

In the control group, the VAS score was significantly lower immediately after surgery (2.83 ± 1.69; *p* < 0.001) and at the end of the follow-up period (0.96 ± 1.08; *p* < 0.001) compared to before surgery (8.17 ± 1.27). The ODI was significantly lower immediately after surgery (0.41 ± 0.12; *p* < 0.001) and at the end of follow-up period (0.23 ± 0.09; *p* < 0.001) compared to before surgery (0.75 ± 0.14). The anterior vertebral body height was 0.73 ± 0.10 before surgery, 0.77 ± 0.09 (*p* < 0.005) immediately after surgery, and 0.72 ± 0.10 (*p* > 0.05) at the end of the follow-up period. The central vertebral body height was 0.70 ± 0.11 before surgery, 0.79 ± 0.07 (*p* < 0.005) immediately after surgery, and 0.75 ± 0.09 (*p* > 0.05) at the end of the follow-up period. The local Cobb angle was 13.94 ± 4.97° before surgery, 10.91 ± 4.91° (*p* < 0.005) immediately after surgery, and 13.55 ± 5.73° (*p* > 0.05) at the end of the follow-up period. By adopting the method of the Bonferroni adjustment, *p* < 0.01 is considered as statistically significant. Therefore, we observed a significant difference (*p* < 0.005) not only between pre- and postoperative, preoperative, and 1‑year follow-up values of VAS and ODI, but also between pre- and postoperative values of anterior vertebral body height, central vertebral body height, and local Cobb angle; however, a minor difference (*p* > 0.05) was observed between preoperative and 1‑year follow-up values of anterior vertebral body height, central vertebral body height, and local Cobb angle. All results for the control group are presented in Tab. 3.

**Tab. 3:** Clinical parameter from initial evaluation to last follow-up in the control group

Parameters	Preop	Postop	1 Month	3 Months	6 Months	1 Year
VAS^a^	8.17 ± 1.27	2.83 ± 1.69	1.31 ± 0.87	0.65 ± 0.86	1.00 ± 0.93	0.96 ± 1.08
ODI	0.75 ± 0.14	0.41 ± 0.12	0.28 ± 0.09	0.23 ± 0.08	0.21 ± 0.08	0.23 ± 0.09
Anterior VB height	0.73 ± 0.10	0.77 ± 0.09	0.75 ± 0.97	0.74 ± 0.10	0.66 ± 0.13	0.72 ± 0.10
Central VB height	0.70 ± 0.11	0.79 ± 0.07	0.79 ± 0.08	0.77 ± 0.08	0.70 ± 0.06	0.75 ± 0.09
Local Cobb angle (°)	13.94 ± 4.97	10.91 ± 4.91	12.47 ± 5.92	12.40 ± 5.63	16.91 ± 7.03	13.55 ± 5.73

*VAS* visual analog scale, *ODI* Oswestry Disability Index, *Preop* preoperative, *Postop* postoperative, *VB* vertebral body

^a^Values represented as mean ± standard deviation

In addition, the difference between the two groups with regard to preoperative VAS (*p* > 0.05), postoperative VAS (*p* > 0.05), VAS at the end of the 1‑year follow-up period (*p* > 0.05), preoperative ODI (*p* > 0.05), and postoperative ODI (*p* > 0.05) were not significant; however, the ODI of two groups at the end of the follow-up period differed significantly (*p* < 0.05). As a result, the study showed that the treatment group had experienced better long-term functional recovery. Furthermore, in both groups, the vertebral body heights and local Cobb angles immediately after surgery were significantly different compared to preoperative vertebral body heights and local Cobb angles. However, the improvement observed in the treatment group was much better than that in the control group, and the deformity correction was sustained until the end of 1‑year follow-up period in the treatment group. While in the control group, the vertebral deformity had returned to the preoperative stage at the end of the 1‑year follow-up period. The results show that the patients in treatment group experienced better and long-lasting bone deformity reduction.

### Perioperative period parameters

The surgical duration was 78.07 ± 13.38 min and 66.29 ± 16.78 min in the treatment and control groups, respectively, which was significantly different (*p* < 0.05). The perioperative blood loss (9.07 ± 5.38 ml and 7.25 ± 3.93 ml in the treatment and control groups, respectively) was significantly different (*p* > 0.05). The cement injection (5.25 ± 1.01 ml and 4.00 ± 1.07 ml in the treatment and control groups respectively) was significantly different (*p* < 0.05). All the parameters were within the range of the operative tolerance of elderly patients (Tab. 4).

**Tab. 4:** Comparison of surgical parameters between treatment and control groups

	Treatment group	Control group
Operative time (min)	78.07 ± 13.38	66.29 ± 16.78
Perioperative blood loss (ml)^a^	9.07 ± 5.38	7.25 ± 3.93
Bone cement injection (ml)^a^	5.25 ± 1.01	4.00 ± 1.07
Patients with bone cement leakage	2	5
Patients with fracture^b^	0	3

^a^Values represented as mean ± standard deviation

^b^Patients who suffered adjacent and nonadjacent vertebra fractures after surgery

### Postoperative complications

A total of 2 patients from the treatment group and 5 patients from the control group experienced cement leakage; however, no related symptoms were recorded. Three patients from the control group had suffered with adjacent and nonadjacent vertebra fractures after surgery: two with adjacent vertebrae fracture and one with both adjacent and nonadjacent vertebra fractures. None of the patients had nerve damage after surgery. There was 1 patient in the control group had requested a walking aid. There was no occurrence of new fracture in surgically treated or adjacent vertebrae in the treatment group (Tab. 4).

## Discussion

PKP is a safe and effective, minimally invasive surgery for the treatment of OVCFs. Theoretically, the technique can be used to correct kyphotic deformities through balloon dilation and injection of cement to stabilize the fractured bone, which ultimately leads to rapid pain relief. Excellent pain relief of PKP had been well documented and it was believed that the immediate pain relief was attributed to immobility and inhibition of micromovement of the fractured fragment after treatment [4, 5]; however, the application of PKP in bone deformity reduction is limited. Another study [6] showed that postural reduction plays an important role in the kyphosis correction rather than the balloon dilation, and it was unrealistic to expect simple PKP to significantly improve the overall spinal deformity [7]. A literature report suggested that deformity, independent of back pain and patient’s age, was a significant factor affecting the functional impairment of these patients [8]. The residual kyphosis after simple PKP may lead to some complications. Adjacent vertebral fracture rates after PKP reported in the literature varied from 6.5–25 % [9–13].

In the present study, the observed rate of nearly 12.5 % was well within this published range. Based on 171 adjacent-segment fractures, it had been proved that in order to avoid subsequent fractures in the same or adjacent level, the vertebral body should be filled adequately and sagittal balance should be obtained with kyphotic angle (KA) correction; balloon kyphoplasty alone could not affect the incidence of subsequent vertebral compression fractures [14]. In a prospective nonrandomized comparative study, Movrin [15] illustrated that altered biomechanics due to local kyphosis was a possible predictive factor for adjacent vertebrae fractures. The subsequent fractures influenced not only the adjacent vertebrae but also the nonadjacent vertebrae [16–19]. Due to the deformity, the spinal load force line would move forward, and this eccentric loading may contribute to the increased new fractures in osteoporotic vertebrae adjacent and nonadjacent to an uncorrected VCF deformity.

The subsequent fractures of adjacent and nonadjacent levels often result in worsening of back pain and spinal deformity, which would impact the quality of life of the patients. Moreover, the increasing forward bending moment requires an increased counterbalancing posterior force from musculature and ligaments, otherwise the balance would be lost. This would cause paraspinal muscle fatigue and contribute to chronic back pain with osteoporotic spinal kyphotic deformity. In the present study, 3 patients from the control group suffered from adjacent or nonadjacent vertebra fractures and 1 patient was unable to walk without crutches because of the posture problems. Hence, in the treatment of OVCFs, deformity reduction is as important as pain relief. Temporary percutaneous pedicle screw reduction would rapidly ease pain as well as effectively correct the kyphotic deformity.

In the present study, the patients in the treatment group had better reduction of OVCF through temporary unilateral pedicle screw reduction, which restored their normal spinal alignment. While in the control group, reduction was not satisfactorily achieved through simple PKP, and the residual kyphosis induced the shift of compressive load path anteriorly to the spine. The ex vivo biomechanical research demonstrated that in the fractured vertebrae, the compressive load path could shift anteriorly by about 20 % of anteroposterior endplate width [20] and it could produce an eccentric loading on the anterior wall. A previous study [21] showed that in anteriorly eccentric compression, peak stresses changed everywhere by less than 11 % and moved to the anterior aspect of the vertebral body. Due to the poor quality of osteoporotic bone, the increasing stress would compress the cancellous bone all the time.

The significant advantage of temporary unilateral pedicle screw reduction with PKP over simple PKP was vertebral height gain even for the patients situation of vertebral compression is worse. In temporary unilateral pedicle screw reduction with PKP, two minimally invasive techniques were involved: first, the temporary percutaneous pedicle screw reduction allowed a more powerful correctional force to achieve better spinal deformity correction, and second the PKP could stabilize the fracture fragment through viscous bone cement to achieve rapid pain relief and durable spinal deformity correction. Temporary unilateral pedicle screw reduction with PKP did not result in further risk and the operative time was about 1.5 h with blood loss of less than 20 ml and with no increase in operative complications compared to simple PKP. These results indicate that TUSR-PKP is safe and effective.

There were concerns regarding TUSR-PKP as to whether the additional procedure of temporary pedicle screw implantation would result in potential complications such as decrease in stiffness and cause extra back pain of adjacent vertebrae. In the present study, the treatment group did not experience any extra back pain or new fracture in the surgically treated or adjacent vertebrae as the temporary unipedicle screw implantation is a minimally invasive procedure, and it would not affect the overall stiffness of the vertebrae body or did not cause extra back pain for at least 1 year. The authors of the present study believe restoring spinal alignment is more important than the local minimal invasiveness. Overall, this retrospective study demonstrated that TUSR-PKP is effective and safe for the treatment of older OVCF patients.

However, the present study has some limitations. The number of patients was limited and this study was not a randomized controlled trial. Hence, a randomized double-blinded prospective study that involves a larger number of patients with long-term follow-up is necessary to confirm the results of this study.

## Conclusion

There is no significant difference between the control group (simple PKP) and the treatment group (TUSR-PKP) with regard to pain relief. Compared to simple PKP, TUSR-PKP has several advantages including fewer subsequent fractures after surgery and better vertebral height gain and ODI improvement, especially in patients whose vertebral compression situation is worse. The initial hypothesis was supported by evidence from the study that TUSR-PKP is noninferior to simple PKP, i.e., it produced at least equal results in managing OVCFs. The authors recommend temporary unilateral pedicle screw reduction with PKP, which is a safe and effective treatment for OVCFs.
